# Crosstalk of carcinoembryonic antigen and transforming growth factor-β via their receptors: comparing human and canine cancer

**DOI:** 10.1007/s00262-015-1684-6

**Published:** 2015-04-02

**Authors:** Erika Jensen-Jarolim, Judit Fazekas, Josef Singer, Gerlinde Hofstetter, Kumiko Oida, Hiroshi Matsuda, Akane Tanaka

**Affiliations:** 1 Department of Comparative Medicine, Comparative Immunology and Oncology, Messerli Research Institute of the University of Veterinary Medicine Vienna, c/o Institute of Pathophysiology and Allergy Research, AKH 4Q, Medical University Vienna and University Vienna, Waehringer Guertel 18-20, 1090 Vienna, Austria; 2 Comparative Animal Medicine, Division of Animal Life Science, Institute of Agriculture, Tokyo University of Agriculture and Technology, Fuchu, Tokyo Japan

**Keywords:** Carcinoembryonic antigen (CEA), CEA-receptor (CEAR), Transforming growth factor beta (TGF-β), Cancer immunology, Regulatory, Nuclear factor kappa-B (NFκB)

## Abstract

There is accumulating evidence that the transforming growth factor beta (TGF-β) and nuclear factor kappa-B (NFκB) pathways are tightly connected and play a key role in malignant transformation in cancer. Immune infiltration by regulatory T- and B-lymphocytes (Tregs, Bregs) has recently gained increased attention for being an important source of TGF-β. There is a plethora of studies examining the pro-tumorigenic functions of carcinoembryonic antigen (CEA), but its receptor CEAR is far less studied. So far, there is a single connecting report that TGF-β also may signal through CEAR. The crosstalk between cancer tissues is further complicated by the expression of CEAR and TGF-β receptors in stromal cells, and implications of TGF-β in epithelial–mesenchymal transition. Furthermore, tumor-infiltrating Tregs and Bregs may directly instruct cancer cells by secreting TGF-β binding to their CEAR. Therefore, both TGF-β and CEA may act synergistically in breast cancer and cause disease progression, and NFκB could be a common crossing point between their signaling. CEAR, TGF-β1–3, TGF-β-R types I–III and NFκB class I and II molecules have an outstanding human–canine sequence identity, and only a canine CEA homolog has not yet been identified. For these reasons, the dog may be a valid translational model patient for investigating the crosstalk of the interconnected CEA and TGF-β networks.

## Introduction

The strategy of comparative oncology is to find homologous molecules, homologous signaling cascades and homologous immune mechanisms to cure cancer in both humans and pets according to the “One Health” principle [1]. Similar to humans, dogs spontaneously develop malignancies with comparable incidence and prevalence and hence represent a natural model for human cancer. For instance, a Swedish study on 80,000 insured female dogs reported that, dependent on higher age and breed, up to 13 % of female dogs had at least one mammary tumor, with an overall-case fatality of 6 % [2]. In humans, females in more highly developed areas have a cumulative risk of 7.1 % of developing mammary cancer by the age of 75, with a mortality rate of 1.7 % [3]. Mammary carcinoma, among others, is thus a burden in both human and veterinary medicines.

The rationale for favouring this tumor entity for comparative studies derives from the fact that it is wise to have access to primary lesions for monitoring tumor progression by caliper measurements. This facilitates the clinical investigations and also takes into consideration that only few centers have access to imaging facilities. Often more than one mamilla are affected in canine cancer patients and may be compared side by side.

It can further be expected that results from comparative oncology studies, investigating naturally occurring cancers due to distinct risk factors in distinct breeds, have a higher translational potential than studies with genetically highly homologous mouse strains [4]. For example, the epidermal growth factor receptor (EGFR) family members EGFR (ErbB1) and human epidermal growth factor receptor 2 (HER-2 (ErbB2)) are molecules of outstanding homology between humans and dogs, and targeting of these molecules results in the same effects on signaling and cancer biology in both species [5, 6].

A more intricate situation was observed for the carcinoembryonic antigen [CEA, also termed carcinoembryonic antigen-related cell adhesion molecule 5 (CEACAM5)], which represents a classical soluble as well as membrane-expressed tumor marker in human clinical oncology. Serum levels of soluble human CEA correlate with disease progression [7], and its assessment is recommended in monitoring the treatment course of colorectal cancer in combination with other prognostic markers [8, 9]. However, CEA molecules are structurally and evolutionarily diverse between humans and canines [10, 11]. A direct CEA homolog in dogs has not yet been defined and represents “a missing link” (Table 1). In contrast, overexpression of CEA in humans has been known for over 20 years to play an important role in metastasis and cell motility [12] by acting as a ligand for E- and L-selectins [13] and might have a signaling function probably by interacting with the Wnt pathway [14]. Furthermore, vaccination with an adeno-associated virus (AAV)–CEA vector combined with Toll-like receptor-9 or Toll-like receptor-7 agonists in wild-type mice resulted in enhanced Th1-mediated immunity and protection from challenge with MC38 colon tumor cells expressing CEA, whereas the same CEA vaccines in CEA transgenic animals promoted tumor growth due to tolerance phenomena elicited by dendritic and myeloid cells [15]. Some CEA family members such as CEACAM6 may adhere to and inhibit tumor-infiltrating cytotoxic T cells [16]. CEACAM1, CEACAM5 and CEACAM6 may be released from epithelial tumors in microvesicles, whereas tumor endothelia only contain CEACAM1 which has a receptor function for other CEACAMs, influences T cell behavior [17] and regulates the tumor matrix and microvascularization [18]. Hence, CEA may affect the tumor and its stroma at the same time [19].

**Table 1:** Interspecies amino acid sequence comparisons

Molecule	Human	Canine	Sequence identity (%)	Sequence similarity (%)
CEAR	HNRPM_HUMAN	XP_005633012.1	99.3	99.5
CEA (CEACAM5)	CEAM5_HUMAN	n.d. [20]	–	–
TGF-β-RI	TGFR1_HUMAN	F1PS63_CANFA	91.8	92.2
TGF-β-RII	TGFR2_HUMAN	F1PNA9_CANFA	87.4	90.3
TGF-β-RIII	TGBR3_HUMAN	F1PIG0_CANFA	88.6	93.0
TGF-β1	TGFB1_HUMAN	TGFB1-CANFA	94.1	96.7
TGF-β2	TGFB2_HUMAN	F1PKH0_CANFA	99.5	99.8
TGF-β3	TGFB3_HUMAN	F1PR85_CANFA	88.4	89.5
NFκB1	NFκB1_HUMAN	NFκB1_CANFA	91.0	94.2
NFκB2	NFκB2_HUMAN	E2RLL2_CANFA	92.3	94.9
RelA	TF65_HUMAN	F1PCU1_CANFA	91.2	93.5

Sequences were from UniProt (http://www.uniprot.org/uniprot/) and from the National Center for Biotechnology Information (NCBI) (http://www.ncbi.nlm.nih.gov/protein). Sequences were aligned using a Needleman–Wunsch algorithm (http://www.ebi.ac.uk/Tools/psa/) with a BLOSUM 62 matrix; gap penalty and end penalty were defined as 10.0 and 0.5, respectively

## CEAR binds TGF-β, a cytokine involved in tolerance induction toward malignant tissue

The scientific history of the carcinoembryonic antigen receptor (CEAR) is much more recent. Interestingly, CEAR showed an outstanding sequence identity of 99 % between the human and canine species [20] (Table 1). The great CEA-receptor homology of humans and dogs on the one hand and the lack of a precise canine CEA equivalent on the other hand are discrepancies and indicate that there could be an alternative ligand. The CEAR was originally described in Kupffer cells and identified as the heterogeneous nuclear ribonucleoprotein M4 (hnRNP M4) [21]. Regarding oncology, it was later also found on colon cancer cells [22]. Moreover, its expression was subsequently also detected in mice in the entire gastrointestinal tract including liver and pancreas [23]. CEAR expression has been connected to inflammation in the liver [24]. In Kupffer cells, a full-length hnRNP M4 (CEARL) and a truncated form (CEARS), generated by alternative splicing, were described [14]. The minimal structural element of human CEACAM5 interacting with hnRNP M4/CEAR was reduced to a peptide of eight amino acids [25].

**Fig. 1:**
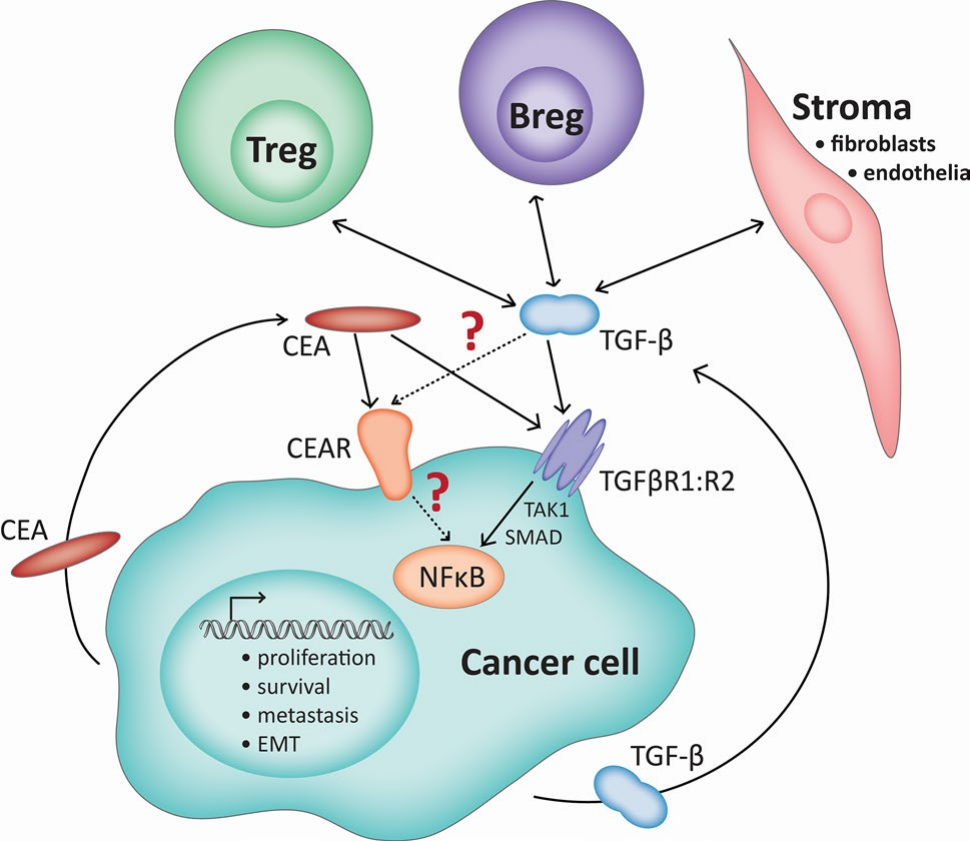
Interconnected networks of CEA and TGF-β signaling in cancer. The cancer cell is an autocrine source of CEA as well as of TGF-β which bind to their specific receptors, CEAR or TGF-β-RI:RII, respectively; the latter signaling via the NFκB pathway. Recently, it has been recognized that CEA also signals via TGF-β-R and initiates the same biological effects [26]. Additionally, Tregs and Bregs, as well as stroma cells, participate in this network by secreting TGF-β. It remains open whether the reverse is the case, and TGF-β may also interfere with the CEAR pathway, which is much less defined

Surprisingly, a recent study has shown that CEA not only signals via its specific receptor, CEAR, but can also bind to the receptor of the important immunomodulatory cytokine transforming growth factor beta (TGF-β, Fig. 1) [26].

## TGF-β sources and its function in the tumor

Three high-affinity membrane-bound receptors for TGF-β are known so far: type I, type II and type III. The classical TGF-β signaling, however, occurs via the heterotetrameric complex of 2 TGF-β-receptor (TGF-β-R) type I and 2 TGF-β-receptor type II transmembrane receptors with serine/threonine kinase activity [27–29]. In the tumor microenvironment, TGF-β is most typically derived from human and canine Foxp3^+^ regulatory T cells (Tregs). It is well known that Tregs can thereby critically dampen anti-tumor immunity and tolerize cytotoxic T cells [30–34]. More recently, intratumoral regulatory B cells (Bregs) have gained attention in human oncology [35, 36]. According to Olkhanud et al. [37], tumor-evoked Bregs should phenotypically resemble activated mature B2 cells (CD19^+^ CD25^hi^ CD69^hi^). Lindner et al. [36] reported that intratumoral Bregs also express granzyme-B (stimulated by IL-21 from Tregs) and a signature of CD19^+^CD38^+^CD1d^+^IgM^+^CD147^+^, as well as including IL-10, CD25 and indoleamine-2,3-dioxygenase. This population seems interesting as a source of TGF-β and for their capacity to suppress intratumoral CD8^+^ and CD4^+^ effector T cells. Bregs can even convert naïve CD4^+^CD25^−^ T cells to Foxp3^+^ Tregs [37]. TGF-β, however, may also be derived from tumor stroma cells [19, 38], where it shapes the microenvironment by interacting with growth factors (epidermal growth factor (EGF), platelet-derived growth factor (PDGF), fibroblast growth factor (FGF), hepatocyte growth factor (HGF), insulin-like growth factor (IGF) [39]), cytokines or chemokines, crosstalking to fibroblasts [40] and supporting the enrichment of endothelial cells, which again shape the extracellular matrix [41]. TGF-β promotes the loss of epithelial markers such as E-cadherin and the accumulation of the mesenchymal marker vimentin in the process of epithelial–mesenchymal transition (EMT) [42]. Importantly, in this case tumor stem cells themselves show an enrichment of mesenchymal markers and are a source of TGF-β. Most studies on EMT are done in mouse or human cancer models [43], but there are reports that EMT transition can be achieved by TGF-β in (normal) Madin–Darby canine kidney (MDCK) cells [44].

Physiologically, TGF-β acts as a tumor suppressor, negatively regulating cellular proliferation, but this is changed in the cancer microenvironment toward a tumor promoter function, where it mediates proliferation, migration, invasion, EMT and metastasis, associated with high miR-181a expression, and altogether termed the TGF-β-paradox [45]. In this context, it is important to note that canines are much closer to the human species than murine animal models. The appearance of Tregs also negatively correlates with prognosis in dog cancer patients [46].

For instance, naive CD4^+^CD25^−^Foxp3^−^ T cells can be converted to Foxp3^+^ Tregs when adoptively transferred into Rag^−/−^ mice only in the presence of TGF-β-positive tumors [47]. Thus, the intratumoral milieu amplifies the cellular sources for even more immunosuppressive cytokines. It has been recently shown that elevated levels of TGF-β and IL-6 in the tumor microenvironment support Th17 cells and that the resulting inflammation was supporting the clinical development and progression of gastric cancer [48]. Although Li et al. have shown that CEA binds to TGF-β-R [26], it has not yet been investigated whether the reverse is true, and TGF-β (besides acting via its own TGF-β-R) may crosstalk via CEAR, thereby imitating the tumor-progressive properties of CEA. CEA modulates effector–target interaction by binding to lymphocytes [49]. Only CEACAM1 expression was previously described in T cells [50], whereas the expression of CEACAM5 on T cells was excluded. Regarding this, we are not aware of investigations on the expression of CEAR on T- or B-lymphocytes.

## TGF-β signaling

In contrast to CEAR, the cellular signaling function of which has to the best of our knowledge not yet been reported, the signaling cascade for the TGF-β-R is well known. The nuclear factor kappa-B (NFκB) is a key master regulator in growth and survival [51, 52]. In normal cells, TGF-β leads to growth inhibition; in short: TGF-β binds to TGF-β-RII, activating TGF-β-RI and then phosphorylating the SMA and MAD homologs SMAD2 and SMAD3, which associate with SMAD4 and together translocate to the nucleus for transcription of genes. All of this is inhibited by SMAD7 [53]. Interestingly, the TGF-β-R-initiated SMAD pathway was shown to target CEACAM5 (and CEACAM6) genes leading to CEA secretion as a mechanism for proliferation in gastric cancer cells [54]. It will be interesting in the future to investigate whether a synergistic crosstalk between the CEA and TGF-β signaling cascades in cancer cells exists.

In human head and neck squamous cell carcinoma cell lines, Freudlsperger et al. [53] could further demonstrate that TGF-β signaling resulted in a sequential phosphorylation of the transforming growth factor-activated kinase-1 (TAK1), inhibitor of nuclear factor kappa-B kinase (IKK), inhibitor of kappa-B subunit alpha (IκBα) and the v-rel avian reticuloendotheliosis viral oncogene homolog A (RelA); however, the crosstalk to CEA was not addressed in this study. Nor did this study address the consecutive activation of TAK1/mitogen-activated protein kinase kinase (MEK)/protein kinase B (AKT)/NFκB and SMAD pathways upon TGF-β stimulation as Gingery et al. [55] did in osteoclasts.

In human cancers, mutations in the TGF-β pathways (e.g., TGF-β-RII or SMAD4) are frequently observed [56]. A recent study has indicated that, although most tested colorectal cancer cells displayed an inactivated TGF-β signaling pathway, they actively secreted TGF-β acting on stromal cells and were thus driving metastasis [57]. In other cancer cell types, TGF-β signaling is intact, but aberrant NFκB activation and NFκB/RelA stimulate proliferation. In this respect, it should be emphasized that NFκB is constitutively activated in a number of hematologic and solid tumors and is one of the major transcription factors associated with cancer progression, inhibition of apoptosis, limitless replicative potential, tissue invasion and metastasis [58].

The TGF-β-R and NFκB pathways are connected via the TAK-1, which (independently, but parallel to SMAD activation) by phosphorylating IKK can directly stimulate the nuclear factor-κB (NFκB) pathway [55]. It is tempting to speculate that CEA may induce similar signals by interacting with TGF-β-R [26]. TAK1 was expressed in head and neck cancers, where nuclear activation of RelA of the NFκB family also took place. TGF-β induced sequential phosphorylation of several targets including TAK1, IKK, IκBα and RelA; additionally, TAK1 again enhanced TGF-β induced NFκB activation [53]. In human neutrophils, a constitutive association of TAK1 and inhibitor of kappa-B (IκB) was recently reported, indicating a close association of these pathways in inflammatory cells [59]. Neil et al. could show that the TAK1-binding protein 1 (TAB 1) forms complexes with IκB kinase b (IKKb) resulting in stimulation of the TAK1:IKKb:RelA pathway. The authors concluded that this axis, including the NFκB elements, is pivotal in the oncogenic transformation of breast cancer [60]. The fact that NFκB plays a critical role in both intrinsic and acquired resistance against endocrine therapy in human breast cancer cells may additionally complicate the situation [61].

## Conclusion

Generally, the dog represents an optimal model organism to study cancer biology in a comparative setting, as many genes represent a great degree of homology to their human counterparts [62]. Even with respect to noncoding RNAs, the significance of similarities between human and dog has recently been acknowledged [63]. Furthermore, the intriguing amino acid homogeneity among human and canine CEAR, TGF-β and TGF-β-R isoforms, NFκB and RelA are given in Table 1, indicating again an advantage of the dog patient in comparative oncology.

We propose that understanding of the crosstalk between CEA and TGF-β signaling toward NFκB as a key cancer regulator, as well as understanding of the Treg and Breg action in tumor tissue, should be extended, possibly with prognostic value. The dog may be a relevant translational model to study these interactions, in line with the comparative oncology strategy [64]. In the future, novel drugs may target the Achilles heel of both obviously interconnected networks.

## Abbreviations

AAV: Adeno-associated virus
Akt: Protein kinase B
Breg: Regulatory B-lymphocyte
CEA: Carcinoembryonic antigen (CEACAM5)
CEACAM: Carcinoembryonic antigen-related cell adhesion molecule
CEAR: Carcinoembryonic antigen receptor
CEARL: Carcinoembryonic antigen receptor, long isoform
CEARS: Carcinoembryonic antigen receptor, short isoform
EGF: Epidermal growth factor
EGFR: Epidermal growth factor receptor (ErbB1)
EMT: Epithelial-to-mesenchymal transition
FGF: Fibroblast growth factor
HER-2: Human epidermal growth factor receptor 2 (ErbB2)
HGF: Hepatocyte growth factor
hnRNP M4: Heterogeneous nuclear ribonucleoprotein M4 (CEAR)
IGFs: Insulin-like growth factors
IKK: Inhibitor of nuclear factor kappa-B kinase
IKKb: Inhibitor of nuclear factor kappa-B kinase subunit beta
IκB: Inhibitor of kappa-B
IκBα: Inhibitor of kappa-B subunit alpha
MDCK: Madin–Darby canine kidney cell line
MEK: Mitogen-activated protein kinase kinase
NFκB: Nuclear factor kappa-B
PDGF: Platelet-derived growth factor
RelA: v-Rel avian reticuloendotheliosis viral oncogene homolog A
SMAD: SMA and MAD homolog
TAB1: TAK1-binding protein 1
TAK1: Transforming growth factor-activated kinase-1
TGF-β: Transforming growth factor beta
TGF-β-R: Transforming growth factor beta receptor
Treg: Regulatory T-lymphocyte
